# High Origin of Radial Arteries: A Report of Two Rare Cases

**DOI:** 10.1100/tsw.2010.187

**Published:** 2010-10-12

**Authors:** Dong Zhan, Yi Zhao, Jun Sun, Eng-Ang Ling, George W. Yip

**Affiliations:** ^1^ Department of Anatomy, Faculty of Basic Medical Sciences, Kunming Medical College, China; ^2^ Department of Anatomy, Yong Loo Lin School of Medicine, National University of Singapore, Singapore

**Keywords:** brachial artery, brachioradial artery, radial artery, ulnar artery, variations

## Abstract

Variations in the arterial supply of the upper limb are relatively common, with reported prevalence rates ranging from 11 to 24.4%. Of these, the most commonly encountered variation in the arm is a high origin of the radial artery. However, after consecutively dissecting and examining 600 Singaporean Chinese cadavers (1,200 upper limbs), we found only two cases of this. In both cases, the brachioradial artery originated from the upper one-third of the brachial artery and continued distally as the radial artery in the forearm. The local prevalence of 0.33% of this variation is significantly lower compared against populations from other geographical regions. Although rare, recognition of the variation is of fundamental importance to clinical practice.

## INTRODUCTION

Variations in the arterial supply of the upper limb are relatively common, with reported frequencies of occurrence ranging from 11 to 24.4%[1]. They can be found at various positions along the axillary, brachial, radial, or ulnar arteries, as well as in the palmar arches[2,3,4,5,6]. Among these, the most common variation in the arm is a high origin of the radial artery from either the axillary or brachial artery. The pattern and rate of occurrence vary by ethnicity. An axillary origin of the radial artery has been reported in 5% of people of African descent compared against 2.7% in the Caucasian population[7]. The prevalence of a high origin of the radial artery from the brachial artery has been reported to lie between 5.9 and 12.1% among Caucasians, but only 2.3% in the Korean population[8,9,10,11]. Although variations in the arterial system of the upper limbs in Asians were studied by Adachi more than 80 years ago[5], the occurrence of a high origin of the radial artery in the Singaporean population has not been reported. In this paper, we present our findings of two cases of this variation after the examination of 1,200 upper limbs in 600 cadavers.

## CASE REPORT

The arterial trees of the upper limbs of 600 Singaporean Chinese cadavers (1,200 upper limbs) were consecutively exposed and examined during routine practical dissections in the undergraduate preclinical medical course by first and second year medical students. Among these, we found only two cases of a high origin of the radial artery, suggesting that the local prevalence of this variation is 0.33%.

The first case was found in the right upper limb of an 86-year-old man with the brachioradial artery originating from the medial side of the upper one-third of the brachial artery and lying posteromedial to it (Fig. 1A). Both arteries ran along the medial side of the humerus posterior to the median nerve. At the cubital fossa, the brachial artery continued as the ulnar artery. The brachioradial artery passed deep to the bicipital aponeurosis and crossed in front of the median nerve, ulnar artery, and biceps brachii tendon to reach the lateral aspect of the forearm, where it continued as the radial artery (Fig. 1A). The remaining courses of the radial and ulnar arteries in the right forearm were normal. No other anomalies were noted in the right upper limb, and no anomalies were seen in the arterial supply of the left upper limb (Fig. 1B).

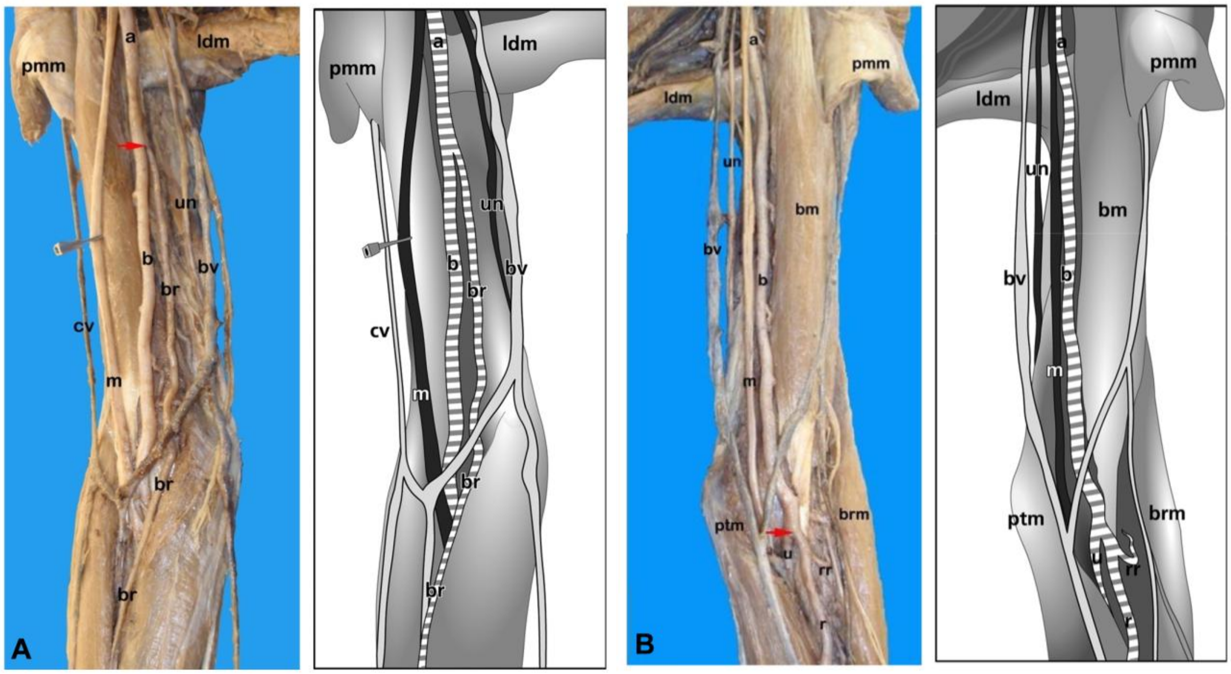

**FIGURE 1.** Anterior view of the arm and forearm of the male cadaver. A brachioradial (br) artery is seen to arise from the brachial artery (b) in the right arm (A). No anomalies are present in the left arm (B). The red arrow indicates the site of origin of the brachioradial artery in (A), and the origin of the radial and ulnar arteries in (B). a, axillary artery; bm, biceps brachii muscle; brm, brachioradialis muscle; bv, basilic vein; cv, cephalic vein; ldm, latissimus dorsi muscle; m, median nerve; pmm, pectoralis major muscle; ptm, pronator teres muscle; r, radial artery; rr, recurrent radial artery; u, ulnar artery; un, ulnar nerve.

The second case involved a 68-year-old woman with a brachioradial artery being present in both her left and right upper limbs (Fig. 2). The course of each brachioradial artery was similar to that described in the male cadaver above. However, the left limb had a much smaller brachioradial artery and the recurrent radial artery originated from the deep brachial artery. No other abnormalities were noted.

In both cases, the diameter of the brachial artery was larger than that of the accompanying brachioradial artery. No median arteries were present. There were no anastomoses with the brachial artery through vessels with a sling-like loop or rectilinear form[12].

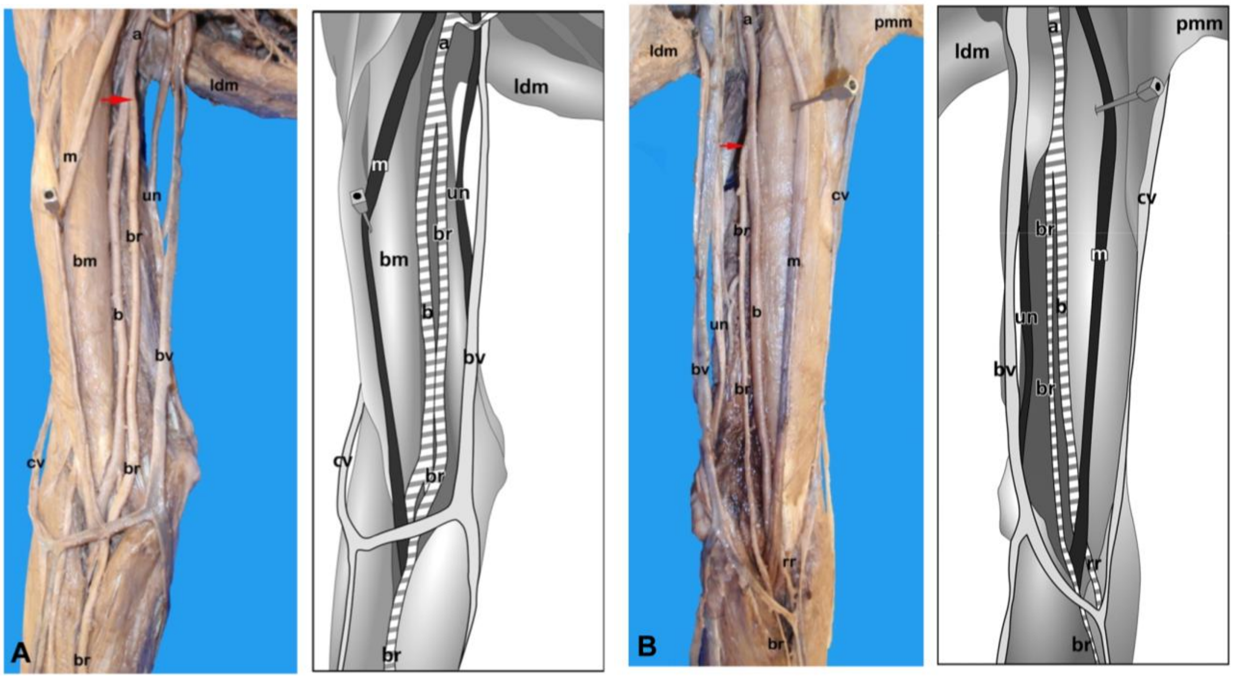

**FIGURE 2.** Anterior view of the arm and forearm of the female cadaver. A brachioradial artery is seen in both the right (A) and left (B) arm and forearm. The red arrow indicates the origin of the brachioradial artery in both panels. Abbreviations as in Fig. 1.

## DISCUSSION

The typical description of the brachial artery in textbooks of human anatomy is that the vessel runs on the medial aspect of the humerus, with the median nerve lying lateral to it initially before crossing over the artery superficially to lie on its medial side at the elbow joint[2,3]. The brachial artery ends at the cubital fossa by giving rise to the radial and ulnar arteries, which supply the forearm.

The brachioradial artery is defined as a radial artery with a high origin[12]. Four types of brachioradial artery have been described, based on its origin from the axillary artery or the upper, middle, or lower third of the brachial artery[12]. It has been suggested that these variations in arterial pattern are the result of persistence, enlargement, or differentiation of capillary plexuses rather than the growth of anomalous vessels[13]. Indeed, the superficial brachial artery is a major artery that is found in the upper limb during vascular development[14]. Persistence of this vessel has been postulated to lead to the formation of the brachioradial artery[11].

To date, the reasons for the different prevalence rates of arterial variations in various geographical regions are not well understood. However, although the brachioradial artery appears to be a relatively rare occurrence among Singaporeans compared against populations from other geographical areas, surgeons, radiologists, and other medical practitioners must be cognizant of its possible presence. Variations in the arterial tree may be encountered during arteriographic examination, percutaneous brachial catheterization, and skin flap elevations from the arm or forearm[1,15,16,17,18,19]. Accidental intra-arterial injections into the brachioradial artery may lead to excessive bleeding, thrombosis, and loss of the upper limb[15,20].

## ACKNOWLEDGMENT

The authors are grateful to S.-L. Bay for technical assistance in drawing diagrams of the dissected specimens.
